# Progress in Diabetes

**Published:** 1902-08-02

**Authors:** 


### Medical Progress.

### Progress in Diabetes.

Herman[1] relates an interesting case of diabetes complicated by pregnancy, and raises the question whether it should not be treated by early induction of labour. As diabetes rarely attacks women under 40 such a complication is rare: the commonest incidence of the disease is between 40 and 60. Kleinwächter states that two-thirds of the cases begin after the menopause. When it begins in child-bearing women, it usually suppresses the menstruation, and sometimes produces atrophy of the uterus; but in a few instances menstruation is unaffected and pregnancy may occur. The disease being severer in the young, the complication of pregnancy usually ensues on a severe type of diabetes. Diabetes may follow pregnancy or pregnancy follow diabetes. In the former, after delivery the sugar generally disappears, though it may return during subsequent pregnancies. Such cases indicate a casual relationship the one with the other. When a diabetic woman has become pregnant the symptoms of the disease have usually become aggravated and its progress hastened. The liquor animi has been found to contain 3 per cent. of sugar, and hydramnios has been observed in an unusual proportion of cases. Premature delivery, due to intra-uterine death, has been observed in two-thirds of published cases. Some cases have proceeded normally, while others have died of collapse and coma with rapid pulse, subnormal temperature, cold extremities, and unconsciousness within a few hours or days of delivery, as after surgical operations.

The dietetic and medicinal treatment of diabetes is not contraindicated during pregnancy, but Herman raises the question in contradistinction to Kleinwächter whether induction of labour is not called for in these cases. The latter says that there is no evidence of its benefit, but Herman says we must act upon probability, and this seems to point to advantage gained by interference.

The symptoms of diabetes, whether favourable or not, are tabulated by Abraham Mayer.[2] Amongst those that are favourable he places: (1) Advanced age at commencement; (2) Long duration of disease without serious complications or much wasting; (3) Traumatic or syphilitic origin; (4) Freedom from the disease of other members of the family; (5) Occurrence in connection with obesity, uric acid, diathesis, or gout; (6) Existence of a mild form of glycosuria with fair tolerance of carbohydrates; (7) Rapid excretion of sugar with increased tolerance of carbohydrates; (8) Favouring conditions of life allowing of thorough dietetic and hygienic regimen; (9) The occurrence of the disease about the time of the menopause. Among the unfavourable symptoms he places (1) Commencement in early life; (2) Early loss of flesh and strength; (3) Severe gastro-intestinal symptoms; (4) Gangrene; (5) Disease coincident in other members of the family; (6) Phthisis, especially with cessation of glycosuria; (7) Large excretion of acetone and increase of ammonia; (8) Unfavourable conditions of life; (9) Organic disease of the pancreas. Mayer considers that some cases of diabetes are associated with an infection of the pancreas by the bacteria of the intestine, and holds that in these cases improvement follows the internal administration of bichloride of mercury. He also believes in the possible contagion of the disease, as in eight per cent. of a large number of cases investigated the husband and wife were both sufferers.

The same author[3] is of the opinion that the chief factor in the causation of coma in diabetics is oxybutyric acid or its derivatives. Indeed, Herter has produced coma in monkeys by injecting 5 per cent. solutions of this acid. Mayer has given glyconic acid to patients and has wondered at the diminution in the urine of the derivatives of the oxybutyric acid, viz., diacetic acid and acetone. He gave a patient in coma 70 grams of glyconic acid neutralised with bicarbonate of soda, and he came out of his comatose state and remained so for three weeks. Coma then ensued, but was relieved for a similar period in the same way. The supply of the drug then became exhausted and the patient died at the third onset.

The dangers of operations on diabetic patients are well known, but Gerster[4] draws attention to the possibility of coma ensuing, even though the amount of sugar present was quite small. Local anæsthesia has been suggested, but on the experience of Fritz-Grossman it would seem there is a special tendency to coma after this.

Loss of power in the ocular muscles is, according to Busby Allen,[5] an early symptom in diabetes: the paralysis is often only partial, with possible intermissions. He states that partial paresis of the accommodation in a person under 35 years of age, and progressing rapidly, should always lead to a suspicion of glycosuria. Optic retinitis of glycosuric origin is a precursor of the grave amblyopia, which is often the precursor of diabetic coma.

In testing for sugar in urine, if the copper solution is turned greenish, further investigation is demanded. Nammack[6] suggests repeated filtration through animal charcoal, which removes all reducing agents except sugar from the urine.

As a result of the analysis of the urines of 56 maternity patients, Carstairs Douglas[7] has found that it is usual for nursing mothers to secrete milk sugar in their urine, at least in the later days of the puerperium. After suckling has stopped, sugar may be found for four or five days later. This fact is of importance with regard to surgical operations or in examinations for life insurance.

The tests for lactose in urine are disappointing, as it is not at all easy to distinguish this from grape sugar. These tests comprise:—(1) Trommer's; (2) Fehling's; (3) Phenylhydrazine, which is definite enough in a watery solution, but very indefinite in urine; (4) the yeast test, which requires time for prolonged action that the lactose may be inverted into glucose and galactose; (5) Barfoed's reagent (a solution of cupric-acetate slightly acidulated with acetic acid), which is reduced by grape sugar but not lactose; if, therefore, the urine does not reduce this reagent until the sugar in it has been inverted by boiling with dilute hydrochloric acid, the presumption is that lactose is present, but this is found not to work well. It is called Ruizard's test; (6) Rubner's colour-test—when 5 c.cm. of sugar solution are boiled with 4 grains of solid neutral lead acetate for two minutes, and ammonia added freely, heat being again applied, a deep-red colour appears in the case of milk sugar; grape sugar yields a chamois-yellow fluid, but the results with urine are disappointing with this test; (7) Nitropropiol. With the last-named, Carstairs Douglas[8] gives his experience. It is the trade name for ortho-nitro-phenyl-propiolic acid. This test depends upon the indigo-blue which is produced on boiling the reagent with a sugar solution. The blue may be rendered apparent when in small quantity by shaking with chloroform which dissolves it. The author has made 200 observations on the delicacy of the test. For glucose the test is more delicate in urine than in aqueous solution—for in water 1-200th grain was detected, while in urine 1-300th was detected. With lactose the recognition is barely possible with a dilution of 1 in 1,500 of water, and the same general results are obtained with urine. Further experiments comparing the yeast and Fehling's tests, prove that nitropropiol gives quite a distinctive reaction where the yeast test fails and the Fehling's solution is doubtful.

The fallacies of the test are sulphides and potassium xanthate, neither of practical importance in testing urine. Albumen, blood and pus, singly or in combination do not interfere. The presence of bile may lead to some doubt, but ammoniacal decomposition and acetone do not interfere. Senna, pot-iodide, chloral hydrate, arsenic, and mercury have each once produced a slight bluish tinge, while some at other times have failed to give it. Also sugar in quantity sufficient to be detected by means of other tests was once indicated where all other tests failed. With these occasional accidents it may be claimed for nitropropiol that though it is affected by other reducing agents than sugar, yet the reaction is striking and distinctive; little urine is required; the test is very delicate; and the reagent is compact, dry, convenient, and comparatively inexpensive.

[1] Edin. Med. Jour., Feb. [2] Med. Rec., Feb. 15. [3] Med. Rec. Dec. 21, 1901. [4] Med. Rec, Feb. 15. [5] Ibid. [6] Ibid. [7] Scottish Med. and Surg. Jour., March. [8] Glasgow Med. and Surg. Jour., Jan.

